# Neurocognitive Profile in Pediatric Kidney Transplant Candidates: Effects of Medical and Sociodemographic Factors

**DOI:** 10.21203/rs.3.rs-4619180/v1

**Published:** 2024-07-18

**Authors:** Lidan Gu, Christopher J Anzalone, Finola Kane-Grade, Danielle Glad, Michael Evans, Sarah Kizilbash

**Affiliations:** University of Minnesota Medical School Twin Cities Campus: University of Minnesota Twin Cities School of Medicine; Boston Children’s Hospital; University of Minnesota Medical School Twin Cities Campus: University of Minnesota Twin Cities School of Medicine; Medical College of Wisconsin; University of Minnesota Clinical and Translational Science Institute: University of Minnesota Twin Cities Clinical and Translational Science Institute; University of Minnesota Medical School Twin Cities Campus: University of Minnesota Twin Cities School of Medicine

**Keywords:** Neurocognitive, kidney failure, neighborhood deprivation, race, dialysis

## Abstract

**Background:**

We evaluated the effects of kidney failure etiology, dialysis, and sociodemographic factors on the subdomains of intellectual functioning in pediatric kidney transplant candidates.

**Methods:**

This retrospective study included 78 pediatric kidney transplant candidates who completed a Wechsler Intelligence Scale assessment during pre-transplant neuropsychological evaluation between 1/1/2010 and 10/31/2022. Linear regression models were employed to examine the effects of kidney failure etiology, dialysis status, neighborhood area deprivation, and race on subdomains of intellectual functioning.

**Results:**

The mean scores of various intellectual functioning domains in pediatric kidney transplant candidates were significantly lower than in the general population (ps <0.001). After adjusting for covariates, patients with congenital anomalies of the kidney and urinary tract had significantly lower processing speed (M=85; 95% CI: 79–91) compared to patients with nephrotic syndrome (M=99; 95% CI: 90–107) and other etiologies (M=84; 95% CI: 78–90) (p=0.003). Patients living in high-level deprivation neighborhoods showed lower working memory performance (M=84, 95% CI: 77–91) than patients living in median-level (M=91, 95% CI: 87–95) and low-level (M=98, 95% CI: 92–104) neighborhood area deprivation (p=0.03). Patients from marginalized racial groups demonstrated lower verbal skills (M=80, 95% CI: 74–87) than White patients (M=92, 95% CI: 88–97) (p=0.02). Additionally, patients receiving dialysis showed higher reasoning skills (M=98, 95% CI: 90–104) than patients without dialysis (M= 90, 95% CI: 86–95) (p=0.04).

**Conclusions:**

Neurocognitive development in pediatric kidney transplant candidates is associated with medical and sociodemographic factors. Strategies to monitor, treat, and accommodate neurocognitive concerns need to be considered to optimize long-term medical and social outcomes.

## Introduction

Childhood intelligence is one of the most powerful predictors of future success[1]. However, typical neurocognitive development is often disrupted for children with complex medical conditions such as kidney failure[2, 3]. Over the past several decades, the survival rates of youths undergoing kidney transplantation have risen, with a 5-year patient survival of 97% in 2020[4]. Despite the great advances in medical treatment to improve survival rates, our understanding of how to support transplant youths to improve neurodevelopmental outcomes remains limited. Current trends indicate that as pediatric kidney transplant recipients enter young adulthood, they are more likely to live with parents, be unemployed, and live without an income when compared to their same-age healthy peers ^[5]^. The suboptimal outcomes and related complications can lead to poor quality of life and substantial costs[6–8].

Recent studies examining the neurocognitive outcomes related to chronic kidney diseases (CKD) and pediatric kidney transplantation have largely focused on identifying the severity of neurocognitive deficits and potential risk factors[3, 9–12]. A systematic review and meta-analysis by Chen and colleagues revealed that a significant proportion of pediatric patients with CKD show intellectual functioning, executive functioning, and academic achievement to be at least one standard deviation below that of the general population, with worse outcomes in patients with advanced CKD, and the poorest outcomes in those requiring dialysis[3]. Furthermore, sociodemographic factors and inequities related to membership in marginalized groups are associated with diminished neurocognitive functions and academic performances[9, 13]. However, both CKD and basic neurocognitive functioning are heterogeneous; therefore, it is unclear how the etiology of kidney failure, sociodemographic factors, and dialysis treatment affect different domains of neurocognitive development. The primary objective of this study was to examine whether and how the underlying etiology of kidney failure, sociodemographic factors, and dialysis are associated with different aspects of intellectual functioning in pediatric kidney transplant candidates.

## Methods

Our retrospective study included a cohort of children and adolescents (ages 3–17) diagnosed with kidney failure and referred for a kidney transplant evaluation at the University of Minnesota/MHealth Fairview between January 2010 and October 2022. All pediatric kidney transplant candidates underwent a pre-transplant neuropsychological evaluation as part of their transplant evaluation. Of the 101 patients who did not opt out of research, 23 patients did not complete a Wechsler Intelligence Scale and were not included in this study. Within these 23 patients, 10 patients (43%) were preschool age between 3 and 5 years; 3 patients (13%) were school-age (6–12 years); and 10 (43%) were adolescents. Nine patients (39%) were from marginalized races. The main reasons for not completing a Wechsler Intelligence Scale included developmental language concerns and lower cognitive functioning that necessitated the use of other cognitive assessments (e.g., The Kaufman Assessment Battery for Children, Universal Nonverbal Intelligence Test, and Leiter International Performance Scale, etc.). The final cohort included 78 patients aged 3–17 years. The Institutional Review Board (IRB) of the University of Minnesota approved this study.

### Outcome Measures

Depending on the age and the year of the neuropsychological testing, patients were administered one of the following: Wechsler Preschool and Primary Scale of Intelligence – 4th Edition (WPPSI-IV), the Wechsler Intelligence Scale for Children – 4th and 5th Editions (WISC-IV and WISC-V, respectively), or the Wechsler Adult Intelligence Scale – 4th Edition (WAIS-IV). Standard scores of subdomains across measures were combined for 1) full-scale IQ (FSIQ), 2) verbal comprehension/skills, 3) perceptual/fluid reasoning, 4) working memory, and 5) processing speed. All Wechsler intelligence scales use standard scores with a mean of 100 and a standard deviation of 15. We defined below-average performance as at least one standard deviation below the normative mean and impaired performance as two standard deviations below the normative mean.

### Medical and Demographic Variables

For the purpose of this study, medical and demographic information was retrieved from the electronic medical records. Medical variables included the etiology of kidney failure, dialysis status, and length of dialysis. The etiologies of kidney failure were grouped into congenital anomalies of the kidney and urinary tract (CAKUT), nephrotic syndrome, and ‘other’ categories. ‘Other’ included nephritis, hemolytic uremic syndrome, cortical necrosis, cystinosis, Wilm’s tumor, cystic kidney disease, nephropathy, Oxalate, Wegener’s Granulomatosis, and kidney failure due to unknown causes. Demographic information included age, sex, race, and neighborhood deprivation index (i.e., ADI). Race included American Indian, Asian, Black, and White. The 2021 area deprivation index (ADI) was collected based on the participant’s 9-digit zip code from the medical chart and Neighborhood Atlas[14]. The ADI was generated based on factors for the theoretical domains of income, education, employment, and housing quality. The index shows the national rank of neighborhood deprivation with higher ADI scores representing greater neighborhood deprivation.

### Data Analysis

Participant characteristics are summarized using frequencies and percentages or means and standard deviations. One-sample t-tests were performed to compare the neurocognitive outcomes across domains of intellectual functioning to the normative mean of the general population (Mean = 100). Neurocognitive outcomes were also compared by etiology of kidney failure, dialysis status, ADI, and race using linear models. These included univariable models that included a single predictor (etiology of kidney failure, dialysis, ADI, or race,). The neurocognitive measures were subsequently analyzed through multivariable models with etiology of kidney failure, dialysis, ADI, race, sex, and age at visit included as predictor variables. Results were summarized using estimated marginal means with 95% confidence intervals. ADI was included in the models as a continuous predictor. The related results were reported as expected values for three representative ADI values (20, 50, and 80) to aid interpretation. Analyses were conducted using R version 4.2.2, including the package emmeans version 1.8.2[15].

## Results

### Participant Characteristics

Table 1 presents participant characteristics. The mean age at kidney failure was 11.28 (SD = 4.03) years. The mean age at the time of neuropsychological assessment was 11.77 (SD = 3.67) years. Within this group, 37 patients (47.4%) were male, and 55 patients (70.5%) were White. Of the 78 patients, 30 (38.5%) were diagnosed with CAKUT, 16 (20.5%) had nephrotic syndrome, and 32 (41%) had ‘other’ causes of kidney failure. Twenty-three (29.5%) patients were on chronic dialysis at the time of neuropsychological assessment. The mean length of dialysis at the time of neuropsychological assessment was 112 days (SD = 374 days). The dialysis status varied by the etiology of kidney failure; only 5 of the 30 patients with CAKUT were on dialysis, only 2 of the 14 patients with nephrotic syndrome were on dialysis, and 18 of the 32 patients with ‘other’ cause of kidney failure were on dialysis at the time of neuropsychological assessment.

### Neurocognitive Profile

**Figure 1:**
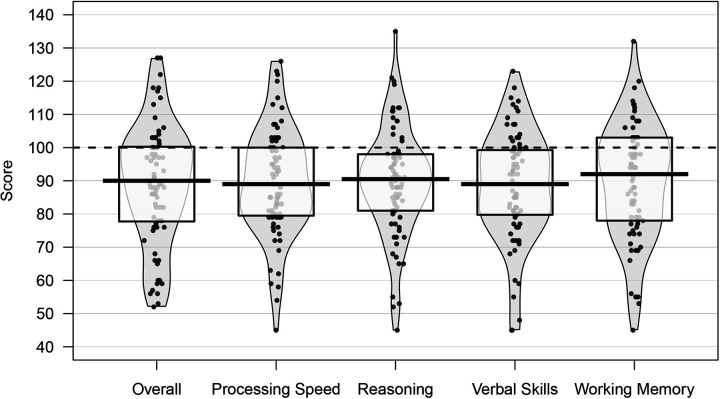
Density plot of neurocognitive outcomes in pediatric kidney transplant candidates

The mean FSIQ score of the entire cohort was 87.42, with a standard deviation of 16.89. Regarding our sample’s mean performances on the subdomains encompassed within FSIQ, the mean standard score of verbal skills was 88.87 (SD = 16.36); reasoning was 92.40 (SD = 16.30); working memory was 90.91 (SD = 17.16); and processing speed was 87.17 (SD = 17.44). As shown in Fig. 1, performance across all domains of intellectual functioning was significantly lower than the normative means (M = 100, SD = 15), with all p-values lower than 0.001.

**Figure 2:**
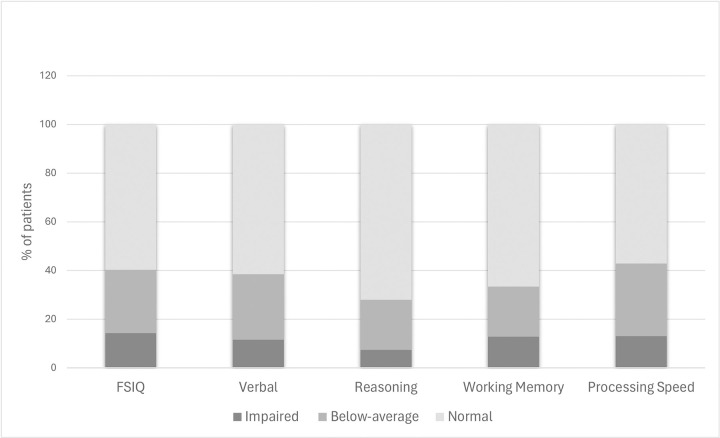
Distribution of below-average and impaired performance in pediatric kidney transplant candidates

As shown in Fig. 2, cognitive difficulties were evident in 31 patients (40.3%) who had FSIQ in the below-average range (i.e., at least one standard deviation below the mean), including 11 patients (14.2%) whose FSIQ was in the impaired range (two standard deviations below the mean). Thirty patients (38.5%) had verbal skills in the below-average range, including 9 patients (11.5%) with impaired verbal skills. Nineteen patients (27.9%) demonstrated below-average performance in the reasoning domain, including 5 patients (7.4%) with impaired performance. Twenty-six patients (33.3%) had working memory performance in the below-average range, including 10 patients (12.8%) who had impaired working memory. Additionally, 33 patients (42.9%) had below-average processing speed, including 10 patients (13%) with impaired processing speed.

### Association between kidney failure etiology and cognitive performance

**Figure 3:**
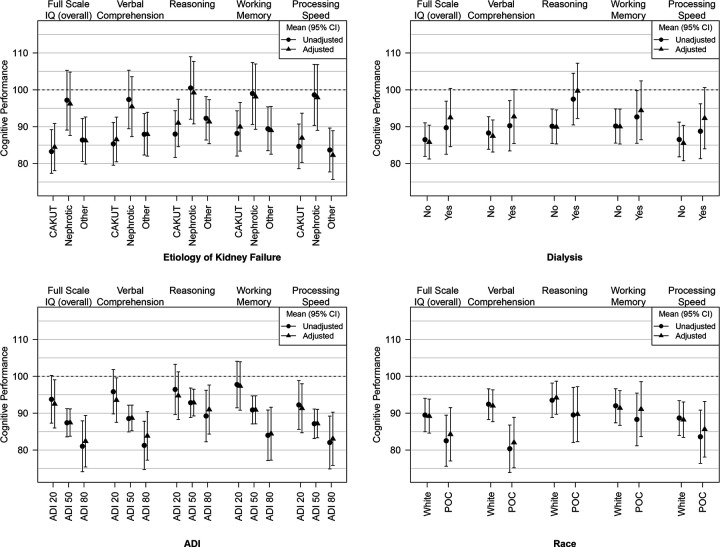
Effects of kidney failure etiology, dialysis, ADI, and race on cognitive performance

On unadjusted analysis, etiology of kidney failure was significantly related to FSIQ (p = 0.02), verbal skills (p = 0.05), and processing speed (p = 0.01). As shown in Fig. 3, patients who had CAKUT had lower mean FSIQ scores (M = 83; 95% CI: 77, 89) compared to patients with nephrotic syndrome (M = 97; 95% CI: 89, 105) and those with ‘other’ as the etiology of kidney failure (M = 86; 95% CI: 81, 92) (p = 0.02). Similarly, patients with CAKUT (M = 85; 95% CI: 80, 91) had lower mean verbal skill scores compared to those with nephrotic syndrome (M = 97; 95% CI:89, 105) and ‘other’ etiology of kidney failure (M = 88; 95% CI: 82, 94) (p = 0.05). Patients with CAKUT also had lower processing speed (M = 85; 95% CI: 79, 91) compared to those with nephrotic syndrome (M = 99; 95% CI: 90, 107) and ‘other’ etiology of kidney failure (M = 84; 95% CI: 78, 90).

After controlling for covariates (dialysis, ADI, race, sex, and age), the etiology remained a significant predictor of the processing speed domain (p = 0.03) (see Table 2). Notably, patients with CAKUT in our sample developed kidney failure at a younger age (M.age = 10.20) compared to patients with nephrotic syndrome (M.age = 13.00). It is also noted that patients with cystic kidney disease (n = 5; M.age = 8.60) had substantially lower mean processing speed (Mean = 67.20, SD = 18.8), with the mean 2 SD lower than the normative mean.

### Association between dialysis and cognitive performance

On unadjusted analysis, dialysis did not appear to have a significant effect on any domain of intellectual functioning, with p-values ranging from 0.09 to 0.63 (see Table 2). However, after controlling for covariates (etiology, ADI, race, sex, and age), dialysis treatment showed a significant and positive impact on performance within the reasoning domain of intellectual functioning (p = 0.04). Patients who did not receive dialysis performed lower on reasoning skills (M = 90, 95% CI: 86, 95) in comparison to patients who received dialysis (M = 98, 95% CI: 90, 104) at the time of neuropsychological assessment.

### Association between ADI and cognitive performance

ADI had a significant effect on FSIQ (p = 0.02), verbal skills (p = 0.006), and working memory (p = 0.01), but not on reasoning (p = 0.21) or processing speed (p = 0.08) on unadjusted analysis. However, after multivariable adjustment, ADI was only significantly associated with working memory (p = 0.03) (see Table 2). Patients who lived in areas with a higher deprivation index showed significantly lower performance on the working memory domain (M = 84, 95% CI: 77, 91) compared to patients who lived in areas with a median level of deprivation index (M = 91, 95% CI: 87, 95) and patients who lived in areas with a low level of deprivation (M = 98, 95% CI: 92, 104).

### Association between race and cognitive performance

On unadjusted analysis, patient race was significantly associated with verbal skills (p = 0.002). However, race was not associated with FSIQ (p = 0.10), reasoning (p = 0.37), working memory (p = 0.39), or processing speed abilities (p = 0.24) (see Table 2). The association between race and verbal skills scores remained significant (p = 0.02) after multivariable adjustment. Patients from marginalized racial backgrounds showed lower performance in the verbal domain (M = 80, 95% CI: 74, 87) compared to White patients (M = 92, 95% CI: 88, 97).

## Discussion

The primary objective of this study was to characterize the effects of the etiology of kidney failure, dialysis treatment, and sociodemographic factors on intellectual functioning in children with kidney failure. We observed variable effects of different factors on the subdomains of intellectual functioning. The etiology of kidney failure had a significant effect on processing speed, while dialysis treatment was significantly associated with reasoning domains. Additionally, we observed a significant association between ADI and working memory, as well as a significant link between race and verbal skills. These findings align with the current literature, suggesting that neurocognitive development may be disrupted in children with CKD. Furthermore, the results indicate that the impact of kidney failure on cognitive development and functioning is multifactorial and can be attributed to various factors, such as the underlying cause, medical treatments, and sociodemographic factors.

The present study expands upon the existing knowledge regarding neurocognitive functioning in pediatric CKD and provides specificity in prediction. Specifically, our findings demonstrate that when accounting for other factors, the etiology of kidney failure is significantly associated with processing speed. Patients who had CAKUT tended to have lower processing speed than patients with nephrotic syndrome. Slower processing speed has been associated with white matter and gray matter loss in some pediatric populations, such as preterm children[16, 17]. White matter loss is also associated with chronic kidney diseases in both pediatric and adult populations[18, 19]. Considering the typical onset of CAKUT during infancy, it is plausible that this condition exerts a more pronounced impact on the change of both white and gray matter compared to other etiologies of kidney failure. However, the difference in age at onset of kidney failure does not fully account for these findings, as our analysis was adjusted for age. Diminished processing speed has been found to be a predictor of reduced adaptive functioning and daily living skills in some pediatric populations[20], highlighting the importance of monitoring and ongoing support for processing speed (e.g., physical therapy, occupational therapy, and educational supports) for patients who have CAKUT and patients who develop kidney failure at an earlier age.

The findings regarding the effect of dialysis treatment on neurocognitive development were mixed. Current literature indicates that the earlier age of dialysis onset and longer time on dialysis are associated with lower neurocognitive outcomes[21–23]. In our study, dialysis emerged as a significant positive contributor only when the analysis was adjusted for other factors in the models. Since most patients with CAKUT were not on dialysis and CAKUT is a significant risk factor for neurocognitive deficits, the high prevalence of CAKUT in the non-dialysis group likely masked the association between dialysis and neurocognitive outcomes, which only became apparent on multivariable analysis. It is also possible that non-dialysis patients performed poorly on the testing due to complications of CKD, such as uremia and anemia, which are often better controlled after dialysis initiation and concomitant initiation of erythropoietin therapy. These findings highlight the potential significance of timely renal replacement therapy in pediatric kidney transplant candidates.

To our knowledge, no other study has examined the relationship between neighborhood deprivation index (i.e., ADI) and intellectual functioning in pediatric kidney transplant candidates. Our study found that neighborhood deprivation negatively predicted working memory performance. Although not specifically using ADI as a proxy for socioeconomic status (SES), a recent meta-analysis of 36 studies confirmed that lower SES is associated with lower working memory performance in the broader pediatric population[24]. Results from this study suggest that pediatric kidney transplant candidates with a socioeconomic disadvantage may benefit from additional tools and strategies to support working memory in educational and medical settings. For instance, providers could use multimodal strategies that emphasize chunking and summarizing information and provide information clearly, concisely, and repeatedly.

The present study demonstrates that race is significantly associated with the verbal comprehension domain. Specifically, White patients tended to perform higher on verbal tasks compared to patients from marginalized racial backgrounds. However, this finding is not surprising given that verbal skills are influenced by social and environmental factors and are, therefore, socially dependent competencies within IQ testing[25, 26]. Furthermore, historically marginalized racial groups experience a lack of access to resources and education to a greater degree than their white counterparts, which could subsequently impact the development of verbal skills in these groups as measured by standardized tests. It is important to consider, though, that usage-based theories purport language and verbal skills in children develop through cultural learning and interaction within a linguistic community[25]. That is, the development of verbal skills is influenced by opportunities for exposure to and use of language within specific cultural contexts for social purposes, which may not be captured in the presently used standardized assessments. Furthermore, 6% of the patients in our sample (5 out of 78) came from families where English was a second language. Individuals whose second language is English have not historically been well represented during measure standardization, which can skew how an individual’s performance is scored compared to same-aged peers. While these findings are important to situate within the broader cultural context and shortcomings of the available cognitive measures, it is still important for providers to be aware of the potential need to facilitate verbal communication and carefully consider early delays in verbal skills for children from marginalized racial backgrounds with kidney failure.

Our study had several limitations. The sample size of this study was modest as pediatric kidney failure is relatively rare[4]. As a result, it was necessary to simplify the categorization of CKD etiology and race. However, this simplification allowed us to unravel the complex interplay of factors influencing neurocognitive development in this vulnerable population. Further, we only included patients who could complete a Wechsler Intelligence Scale to allow further analysis of specific domains within intellectual functioning. Patients who were not able to complete a Wechsler Intelligence Scale in our sample were younger (i.e. preschool age), had language difficulties, or had less developed cognitive functioning. The single-center nature of this study is also a limitation. Future research should explore a multi-site design to increase the sample size and account for variations in abilities to broaden the generalizability of these findings to a broader population. Such studies would provide a more comprehensive understanding of the neurocognitive development in pediatric kidney failure and help validate the findings across different healthcare settings. This research design would also allow the exploration of additional medical variables such as the duration of illness or the age onset for kidney failure and possibly allow for further characterization of the associations between brain structure change, processing speed, and adaptive functioning in children with kidney failure.

## Conclusion

Neurocognitive development in pediatric kidney transplant candidates is often disrupted. The current study found that the etiology of kidney failure, neighborhood deprivation level, and race play a role in neurocognitive development in the domains of processing speed, working memory, and verbal skills, respectively. Thus, it is important to consider monitoring and providing accommodations in both educational and medical settings for working memory, processing speed (for patients who have CAKUT and patients who develop kidney failure at an earlier age), and verbal communication (e.g., speech/language therapy services, particularly among children from marginalized racial backgrounds).

## Figures and Tables

**Table 1:** Participant Characteristics

Variable	Overall
n	78
Sex = Male (%)	37 (47.4)
Race Category (%)	
White	55 (70.5)
American Indian or Alaska Native	10 (12.8)
Asian	7 (9.0)
Black or African American	6 (7.7)
Insurance category = Public (%)	40 (51.3)
Age (mean (SD))	11.77 (3.67)
Kidney Failure Etiology (%)	
CAKUT	30 (38.5)
Nephrotic syndrome	16 (20.5)
Other	32 (41.0)
Dialysis = Yes (%)	23 (29.5)
Dialysis length (mean (SD))	112.11 (373.76)
Full Scale IQ (mean (SD))	87.42 (16.89)
Verbal Skills (mean (SD))	88.87 (16.36)
Reasoning (mean (SD))	92.40 (16.30)
Working Memory (mean (SD))	90.91 (17.16)
Processing Speed (mean (SD))	87.17 (17.44)

**Table 2:** Neurocognitive measure comparisons based on medical cause, race, and dialysis

	Overall IQ			
	unadjusted		adjusted	
	mean (95% CI)	p-value	mean (95% CI)	p-value
**Kidney failure etiology**				
CAKUT	83 (77, 89)	0.02	84 (77, 92)	0.09
Nephrotic syndrome	97 (89, 105)		96 (88, 105)	
Other	86 (81, 92)		86 (80, 93)	
**Dialysis**				
No	86 (82, 91)	0.45	86 (81, 90)	0.17
Yes	90 (82, 97)		92 (85, 100)	
**Area Deprivation**				
20	94 (87, 100)	0.02	92 (86, 99)	0.08
50	87 (84, 91)		88 (84, 91)	
80	81 (74, 88)		82 (75, 89)	
**Race**				
Person of Color	82 (76, 90)	0.10	84 (77, 92)	0.28
White	90 (85, 94)		89 (85, 94)	
	Verbal Skills			
	unadjusted		adjusted	
	mean (95% CI)	p-value	mean (95% CI)	p-value
**Kidney failure etiology**				
CAKUT	85 (80, 91)	0.05	86 (80, 93)	0.21
Nephrotic syndrome	97 (89, 105)		95 (87, 104)	
Other	88 (82, 94)		88 (82, 94)	
**Dialysis**				
No	88 (84, 93)	0.63	88 (83, 92)	0.25
Yes	90 (83, 97)		93 (85, 100)	
**Area Deprivation**				
20	96 (90, 102)	0.006	94 (88, 100)	0.07
50	88 (85, 92)		89 (85, 92)	
80	81 (75, 88)		84 (77, 90)	
**Race**				
Person of Color	80 (74, 87)	0.002	82 (75, 89)	0.02
White	92 (88, 97)		92 (88, 96)	
	Reasoning			
	unadjusted		adjusted	
	mean (95% CI)	p-value	mean (95% CI)	p-value
**Kidney failure etiology**				
CAKUT	88 (82, 94)	0.07	91 (85, 97)	0.26
Nephrotic syndrome	100 (92, 109)		99 (91, 108)	
Other	92 (86, 98)		91 (85, 97)	
**Dialysis**				
No	90 (86, 95)	0.09	90 (85, 95)	0.04
Yes	98 (90, 104)		100 (92, 107)	
**Area Deprivation**				
20	96 (90, 103)	0.21	95 (88, 101)	0.49
50	93 (89, 97)		93 (89, 96)	
80	89 (82, 96)		91 (84, 98)	
**Race**				
Person of Color	90 (82, 97)	0.37	90 (82, 97)	0.33
White	94 (89, 98)		94 (90, 99)	
	Working Memory			
	unadjusted		adjusted	
	mean (95% CI)	p-value	mean (95% CI)	p-value
**Kidney failure etiology**				
CAKUT	88 (82, 94)	0.10	90 (83, 97)	0.24
Nephrotic syndrome	99 (91, 107)		98 (89, 107)	
Other	89 (84, 95)		89 (83, 96)	
**Dialysis**				
No	90 (86, 95)	0.57	90 (85, 95)	0.38
Yes	93 (86, 100)		94 (86, 102)	
**Area Deprivation**				
20	98 (92, 104)	0.01	97 (91, 104)	0.03
50	91 (87, 95)		91 (87, 95)	
80	84 (77, 91)		84 (77, 92)	
**Race**				
Person of Color	88 (81, 95)	0.39	91 (84, 99)	0.95
White	92 (87, 97)		91 (87, 96)	
	Processing Speed			
	unadjusted		adjusted	
	mean (95% CI)	p-value	mean (95% CI)	p-value
**Kidney failure etiology**				
CAKUT	85 (79, 91)	0.01	87 (80, 94)	0.03
Nephrotic syndrome	99 (90, 107)		98 (89, 107)	
Other	84 (78, 90)		82 (76, 89)	
**Dialysis**				
No	86 (82, 91)	0.61	86 (81, 90)	0.19
Yes	89 (81, 96)		92 (84, 101)	
**Area Deprivation**				
20	92 (86, 99)	0.08	91 (85, 98)	0.16
50	87 (83, 91)		87 (83, 91)	
80	82 (75, 89)		83 (76, 90)	
**Race**				
Person of Color	84 (76, 91)	0.24	86 (78, 93)	0.58
White	89 (84, 93)		88 (83, 93)	

**Note**:

Unadjusted model: univariable

Adjusted model: adjusted for race, cause of kidney failure, dialysis, area deprivation index, sex, and age at visit

## Abbreviations

CKD: Chronic Kidney Diseases
CAKUT: Congenital Anomalies of the Kidney and Urinary Tract ADI: Area Deprivation Index
FSIQ: Full Scale Intelligence Quotient
